# Prevention, incidence, and risk factors of chyle leak after radical nephrectomy and thrombectomy

**DOI:** 10.1002/cam4.6858

**Published:** 2023-12-20

**Authors:** Kewei Chen, Zhuo Liu, Yuxuan Li, Xun Zhao, Guoliang Wang, Xiaojun Tian, Hongxian Zhang, Lulin Ma, Shudong Zhang

**Affiliations:** ^1^ Department of Urology Peking University Third Hospital Beijing P.R. China

**Keywords:** chyle leak, radical nephrectomy, renal cell carcinoma, tumor thrombus

## Abstract

**Background:**

To define the incidence and risk factors of chyle leak (CL) after radical nephrectomy and thrombectomy and to determine the impact of chyle leak on oncological outcomes.

**Patients and Methods:**

A total of 445 patients who underwent radical nephrectomy and thrombectomy between January 2014 and January 2023 were included. CL is defined as the drainage of chyle with a triglyceride level greater than 110 mg/dL after oral intake or enteral nutrition. Multivariate logistic regression analysis was performed to identify the risk factors of postoperative (CL). The Kaplan–Meier curves were used to compare overall survival and cancer‐specific survival.

**Results:**

44 patients (9.9%) were diagnosed as (CL). All patients developed CL within 6 days after the operation with a median time of 3 days. In multivariate logistic regression analysis, Mayo grade and side were independent patient‐related risk factors. In addition, operation approach, operation time, and number of lymph nodes harvested were independent surgery‐related risk factors. Between the CL group and the non‐CL group, neither overall survival nor cancer‐specific survival showed statistical differences.

**Conclusion:**

Based on this retrospective study of renal cell carcinoma and tumor thrombus patients in our center, we found that the risk factors were Mayo grade, side, operation approach, operation time, and number of lymph nodes harvested, and the occurrence of CL significantly prolonged hospital stay, but had no effect on long‐term oncological outcomes.

## 1 INTRODUCTION

Chyle leak (CL) is a rare but clinically relevant complication of urological surgeries that involves the direct disruption of the cisterna chyli or its tributaries.^1^, ^2^, ^3^ The cisterna chyli is a lymphatic sac located next to the abdominal aorta and inferior vena cava (IVC) that collects chyle from the intestines.^3^ CL after surgery is defined by most clinicians as the drainage of chyle with a triglyceride level greater than 110 mg/dL after oral intake or enteral nutrition.^4^, ^5^ Sustained loss of chyle may result in hypoalbuminemia, lymphocytopenia, and electrolyte imbalance,^6^ which may increase the mortality risk in severe cases.^7^ Furthermore, several studies have shown that postoperative CL is associated with a higher probability of peritoneal injury and worse oncological outcomes.^4^, ^7^

The incidence of CL in open or laparoscopic nephrectomy is approximately 0.77% to 5.9% according to current literature.^2^, ^3^, ^8^, ^9^ Radical nephrectomy and thrombectomy (RNAT) is the standard surgical treatment for renal cell carcinoma (RCC) with tumor thrombus (TT) invading the IVC. RNAT increases the 5‐year tumor‐specific survival rate to 40% to 65%,^10^ but it also increases the risk of CL compared to radical nephrectomy alone considering the anatomical proximity of the IVC to the lymphatic ducts or cisterna chyli.^6^ However, only few studies have demonstrated the incidence, prevention, and risk factors of CL and its correlation with prolonged hospitalization in patients with RCC and TT.^3^ In addition, there is no literature that clarifies the impact of postoperative CL on the long‐term outcome of patients with RCC and TT.

The aim of this study was to define the incidence and risk factors of CL after RNAT in a setting with early oral intake and to determine the impact of CL on postoperative hospitalization. A further objective was to assess the impact of CL on oncological outcomes.

## 2 PATIENTS AND METHODS

This study is a retrospective analysis based on the database of RCC with TT in our medical center. The clinicopathological data of 487 patients who underwent RNAT between January 2014 and January 2023 were collected, and the cases included in this study meet the following criteria. The inclusion criteria were as follows: 1. radical nephrectomy and thrombectomy; 2. preoperative imaging showed that the tumor invaded the renal vein or IVC; 3. age above 18 years old; and 4. postoperative histopathological data were available. The exclusion criteria were as follows: 1. concurrent resection of distant metastasis; 2. emergency surgery; 3. death within 7 days after surgery; and 4. unavailable clinicopathological data. A total of 445 patients were included in this study after screening. This study was conducted in accordance with the ethical standards of the Helsinki Declaration and was approved by our local ethics committee.

The definition of CL after RNAT is as follows: milky‐white drainage outflow with triglyceride concentration greater than 110 mg/dL.^4^, ^11^ If CL is suspected after the drainage tube removal, ultrasound‐guided abdominal paracentesis is performed to confirm the diagnosis of CL. The onset time of postoperative CL was defined as the time interval from surgery to the appearance of CL. The time of CL resolution was defined as the time interval between appearance of CL and negative triglyceride level in drainage fluid. The time of oral intake was defined as the interval between the confirmed appearance of CL and the first oral intake of more than 500 mL of drinking water. According to the CL grade proposed by van der Gaag,^12^ Grade A was defined as CL lasting less than 7 days, Grade B was defined as CL resolved within 7–14 days, and Grade C was defined as CL resolved more than 14 days or requiring surgical intervention. Overall survival (OS) is defined as the time from the date of RNAT to death due to any reason or the last follow‐up. Cancer‐specific survival (CSS) was defined as the time from the date of RNAT to the patient's death due to the primary tumor or the last follow‐up.

### 2.1 Surgical treatment

According to the patient's tumor side and TT height, different surgical methods were adopted,^13^, ^14^ the principle is kidney first and thrombus last. For Mayo 0 TT, the surgical technique is the same as radical nephrectomy. For Mayo I or II TT, the thrombus is removed by the balloon catheter method if TT does not invade the vessel wall of IVC. If TT is seriously adhered to the IVC or invaded IVC, different surgical methods are adopted based on the side of the tumor. When tumor is located on the left, the left renal artery is embolized first. After the kidney was separated, the left renal vein was cut off and the distal end of IVC, the right renal artery, the right renal vein, and the proximal end of IVC were blocked in sequence. Then TT was removed and IVC was reconstructed finally. When the tumor is located on the right, the right renal artery is blocked and cut first, and then the distal end of IVC, the left renal vein, and the proximal end of IVC are blocked in sequence. Then TT was removed and IVC was reconstructed finally. For Mayo III TT, the liver ligament was severed to separate the liver from the diaphragm upstream. The main hepatic vein was blocked first, and then the proximal IVC was blocked. After the removal of IVC TT above the vena porta and the reconstruction of IVC was completed, the IVC below the vena porta area was blocked to complete the resection of TT and reconstruction of IVC below the vena porta area, which is to shorten the liver ischemia time. For Mayo IV TT, the central tendon of the diaphragm was cut around the vena cava, or the diaphragm was directly cut and then TT was gently pushed into the IVC to change it to the lower diaphragm in order to further remove the TT.

### 2.2 Statistical analysis

Statistical analysis was performed by R version 3.6.1 for Mac. Continuous variables were expressed as mean ± SD and categorical variables were expressed as percentages. Continuous data were compared between groups using Student's *t*‐test (data conforms to normal distribution) and Mann–Whitney U (data does not conform to normal distribution). Categorical data were compared between groups by Chi‐square test and Fisher exact test. Univariate and multivariate logistic regression analyses were performed to identify the risk factors of postoperative CL. Pairwise analysis was performed using the propensity score matching (PSM) method with a 1:1 pairing. The Kaplan–Meier curves were used to compare OS and CSS between two groups and differences among groups were tested by log‐rank. *p* < 0.05 was considered statistically significant.

## 3 RESULTS

**FIGURE 1:**
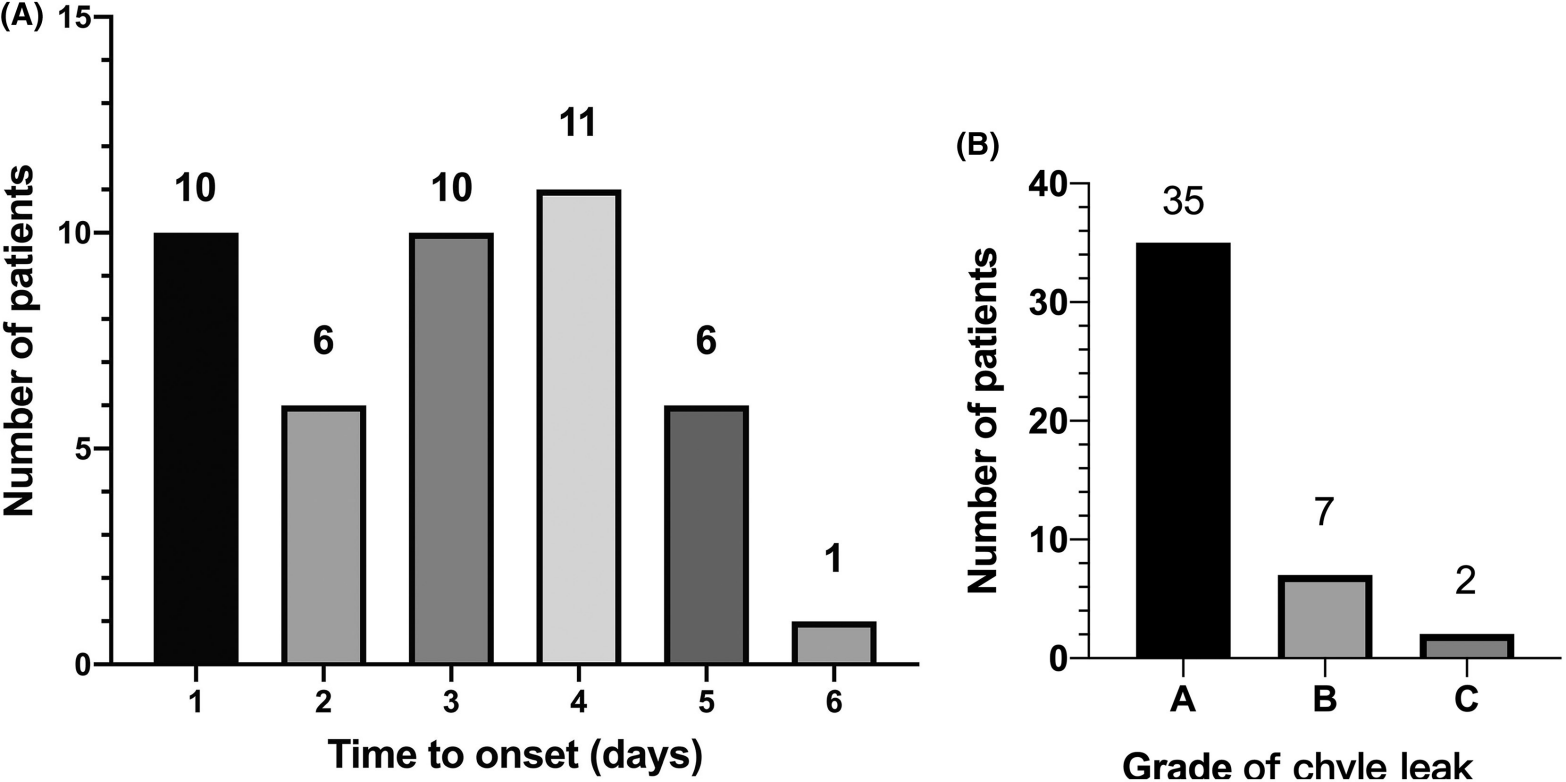
(A) Time distribution of patients with postoperative chyle leakage. (B) Grade distribution of patients with postoperative chyle leakage.

**FIGURE 2:**
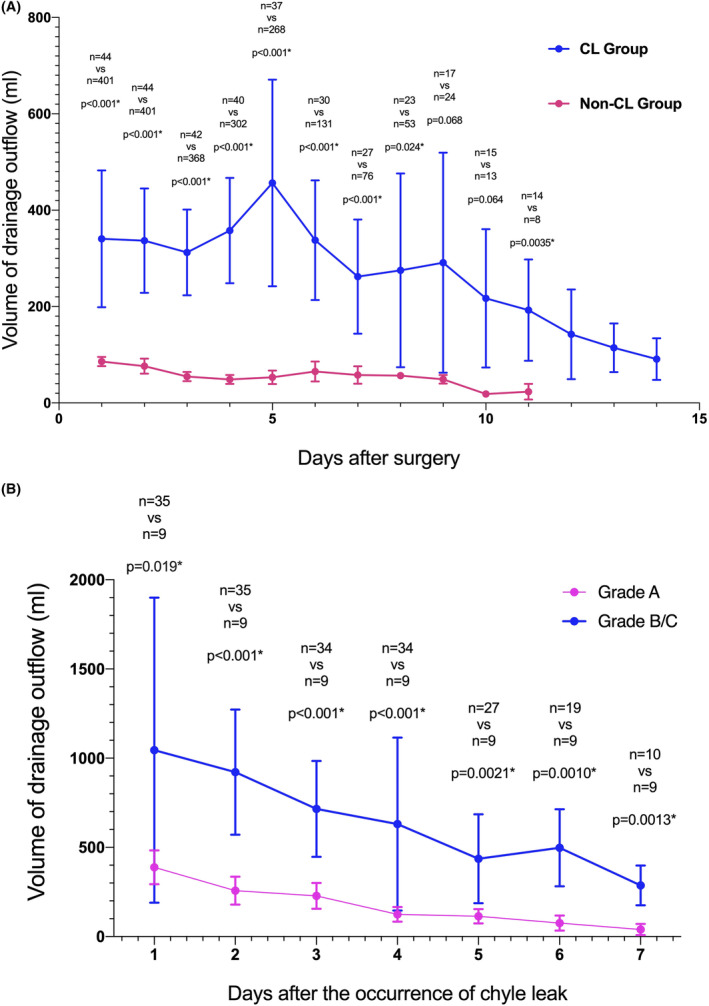
(A) The drainage volume of chyle leak patients and non chyle leak patients after surgery. (B) The drainage volume of Grade A patients and Grade B/C patients after the occurrence of chyle leak.

A total of 445 patients who received RNAT from January 2014 to January 2023 were included in this study and 44 patients (9.9%) were diagnosed as CL. 27 out of 44 patients with CL underwent lymphadenectomy, and 180 out of 401 patients without CL also underwent lymphadenectomy (87.0% vs. 13.0%, *p* = 0.038). All patients developed CL within 6 days after the operation with a median time of 3 days, and most patients (84.1%) developed CL within 4 days after operation (Figure 1A). According to the classification of CL proposed by van der Gaag,^12^ we divided the patients with CL into Grade A, Grade B, and Grade C, with 35 patients, 7 patients, and 2 patients, respectively (Figure 1B). The drainage volume of patients with lymphatic leakage significantly increased (Figure 2A,B). We summarized the clinicopathological data of patients in Table 1 and compared the data between patients with CL and non‐CL.

**TABLE 1:** Patient demographic and clinicopathological data.

	Total (*n* = 445)	Chyle leak group (*n* = 44)	Non‐chyle leak group (*n* = 401)	*p* value
Gender				0.85
Male	308 (69.2%)	31 (70.5%)	277 (69.1%)	
Female	137 (30.8%)	13 (29.5%)	124 (30.9%)	
Age (years)	58.3 ± 11.8 (20–83)	56.8 ± 12.7 (23–78)	58.5 ± 11.7 (20–83)	0.40
Weight (kg)	67.6 ± 12.0 (37.0–115.0)	65.3 ± 12.9 (43.5–100.0)	67.8 ± 11.9 (37.0–115.0)	0.21
Height (cm)	166.9 ± 7.6 (143.0–188.0)	166.1 ± 7.5 (150.0–180.0)	167.0 ± 7.60 (143.0–188.0)	0.48
Body Mass Index (kg/m^2^)	24.1 ± 3.59 (15.2–39.0)	23.5 ± 3.76 (16.7–33.0)	24.2 ± 3.57 (15.2–39.0)	0.27
Operation approach				0.006*
Open	199 (44.7%)	26 (59.1%)	173 (43.1%)	
Laparoscopy	169 (38.0%)	7 (15.9%)	162 (40.4%)	
Robot‐assisted laparoscopy	77 (17.3%)	11 (25.0%)	66 (16.5%)	
Laparoscopy to open				0.22
Yes	47 (10.6%)	7 (15.9%)	40 (10.0%)	
No	398 (89.4%)	37 (84.1%)	361 (90.0%)	
Mayo grade				0.006*
0 or 1	197 (44.3%)	11 (25.0%)	186 (46.4%)	
2, 3 or 4	248 (55.7%)	33 (75.0%)	215 (53.6%)	
Ipsilateral adrenalectomy				0.35
Yes	223 (50.1%)	25 (56.8%)	198 (49.4%)	
No	222 (49.9%)	19 (43.2%)	203 (50.6%)	
Partial resection of IVC				0.73
Yes	112 (25.2%)	12 (27.3%)	100 (24.9%)	
No	333 (74.8%)	32 (72.7%)	301 (75.1%)	
Lymph node dissection				0.0006*
Yes	195 (43.8%)	30 (68.2%)	165 (41.2%)	
No	250 (56.2%)	14 (31.8%)	236 (58.8%)	
Lymph node harvest	4.8 ± 4.1 (*n* = 195, range, 1–31)	6.3 ± 6.0 (*n* = 30, range, 1–31)	4.5 ± 3.6 (*n* = 165, range, 1–20)	0.040*
Hemoglobin (g/L)	123.5 ± 23.6 (40–232)	115.9 ± 18.7 (84–149)	124.3 ± 23.9 (40–232)	0.011*
Albumin (g/L)	39.4 ± 5.4 (19–51)	38.1 ± 6.2 (21–50)	40.0 ± 5.3 (19–51)	0.17
Preoperative Scr (μmol/L)	102.5 ± 79.1 (32–958)	99.4 ± 43.6 (45–304)	102.8 ± 82.1 (32–958)	0.86
postoperative Scr (μmol/L)	113.8 ± 81.0 (34–875)	120.5 ± 75.8 (51–445)	113.0 ± 81.6 (34–875)	0.55
Side				0.0001*
Left	175 (39.3%)	29 (65.9%)	146 (36.4%)	
Right	270 (60.7%)	15 (34.1%)	255 (63.6%)	
Tumor diameter (cm)	8.7 ± 3.3 (1.5–21.1)	9.3 ± 3.8 (4.0–21.1)	8.7 ± 3.3 (1.5–20.0)	0.40
Hepatic vein invasion				0.52
Yes	7 (1.6%)	1 (2.3%)	6 (1.5%)	
No	438 (98.4%)	43 (97.7%)	395 (98.5%)	
Perinephric tissues invasion				0.057
Yes	136 (30.6%)	19 (43.2%)	117 (29.2%)	
No	309 (69.4%)	25 (56.8%)	284 (70.8%)	
Sarcomatoid differentiation				0.27
Yes	68 (15.3%)	4 (9.1%)	64 (16.0%)	
No	377 (84.7%)	40 (90.9%)	337 (84.0%)	
Pathological type				0.20
Clear cell carcinoma	300 (67.4%)	28 (63.6%)	272 (67.8%)	
Papillary cell carcinoma	39 (8.8%)	7 (15.9%)	32 (8.0%)	
Other	106 (23.8%)	9 (20.5%)	97 (24.2%)	
ASA classification				0.70
1	24 (5.4%)	2 (4.5%)	22 (5.5%)	
2	354 (79.6%)	34 (77.3%)	320 (79.8%)	
3	61 (13.7%)	8 (18.2%)	53 (13.2%)	
4	6 (1.3%)	0 (0%)	6 (1.5%)	
Operation time (min)	305.6 ± 134.8 (60–995)	373.5 ± 185.5 (91–1114)	298.2 ± 126.1 (60–995)	0.0036*
Intraoperative hemorrhage (mL)	1056 ± 1373 (5–10,000)	1694 ± 1730 (10–7000)	985.8 ± 1312 (5–10,000)	0.0008*

Abbreviations: ASA, American Society of Anesthesiologists; IVC, inferior vena cava; Scr, serum creatine.

^*^ Means p<0.05.

### 3.1 Risk factors of CL

**FIGURE 3:**
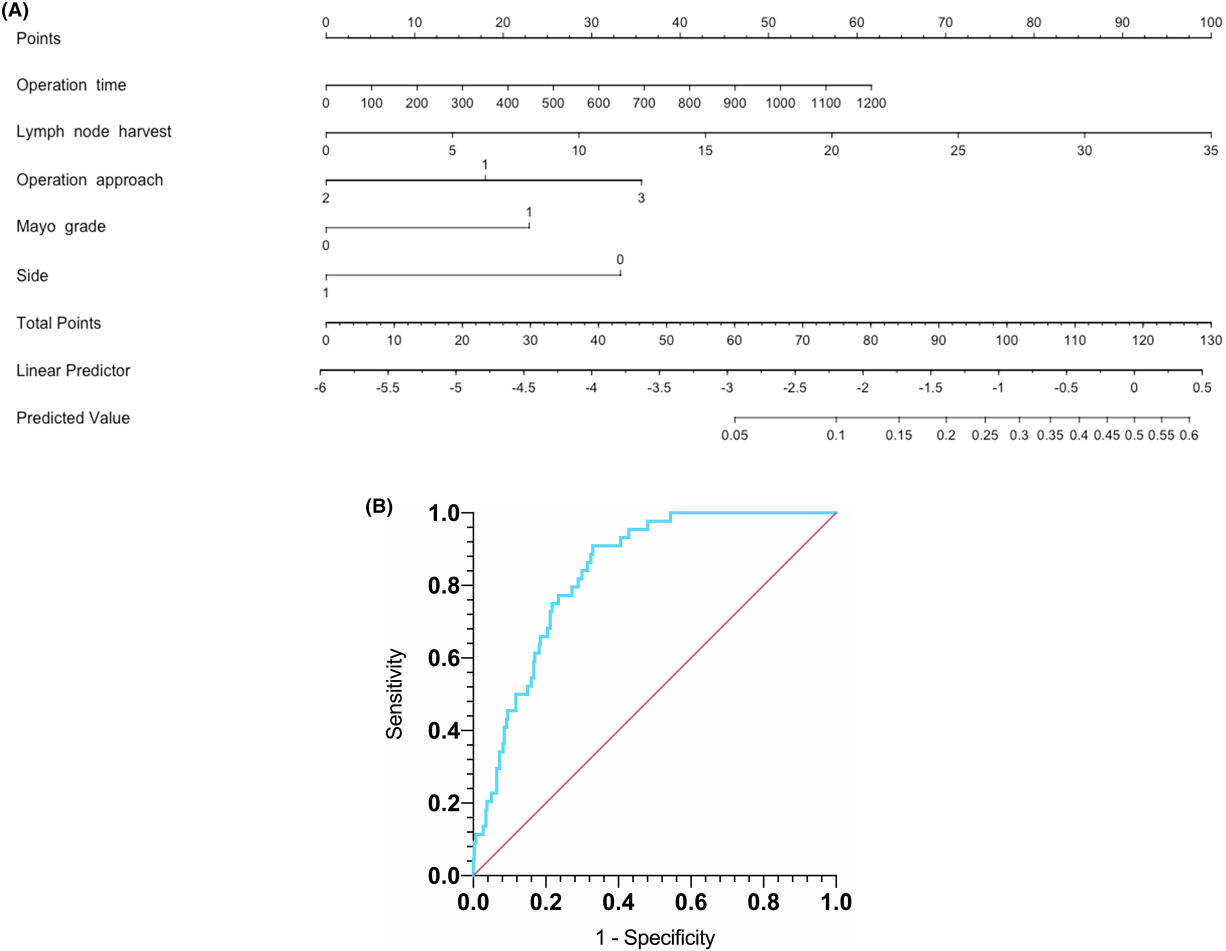
(A) A nomogram predicting the probability of postoperative chyle leak after radical nephrectomy and thrombectomy. (B) Receiver operating characteristics curve for the nomogram. The area under the ROC curve is 0.837, the 95% CI is 0.790–0.884 and *p* < 0.0001.

In multivariate logistic regression analysis, Mayo grade (OR 3.181; 95% CI 1.319–8.201; *p* = 0.013) and side (OR 0.1863; 95% CI 0.08385–0.3940; *p* < 0.0001) were independent patients‐related risk factors. In addition, operation approach (OR 1.764; 95% CI 1.239–2.531; *p* = 0.0017), operation time (OR 1.003; 95% CI 0.9999–1.005; *p* = 0.042) and number of lymph nodes harvest (OR 1.160; 95% CI 1.080–1.246; *p* < 0.0001) were independent surgery‐related risk factors. The results of univariate and multivariate logistic regression analysis are summarized in Table 2. Based on the above factors, a predictive nomogram for postoperative CL was established. As shown in Figure 3A, the higher the total score, the higher the probability of CL long‐term regression. As shown in Figure 3B, the area under AUC curve was 0.837.

**TABLE 2:** Univariate and multivariate logistic regression analysis of patients with chyle leak after radical nephrectomy and thrombectomy.

Variables	Univariate analysis			Multivariate analysis		
	OR	95% CI	p	OR	95% CI	p
Operation approach	1.431	1.089–1.867	0.0088*	1.764	1.2^*^39–2.531	0.0017*
Mayo grade (0 or 1 vs. 2, 3 or 4)	2.595	1.314–5.511	0.0085*	3.181	1.319–8.201	0.013*
Side	0.2961	0.1501–0.5623	0.0003*	0.1863	0.08385–0.3940	<0.0001*
Operation time	1.003	1.001–1.005	0.0009*	1.003	0.9999–1.005	0.042*
Intraoperative hemorrhage	1.000	1.000–1.000	0.0023*	1.000	0.9998–1.000	0.91
Hemoglobin	0.9856	0.9724–0.9988	0.033*	0.9880	0.9725–1.004	0.13
Lymph node harvest	1.134	1.061–1.213	0.0002*	1.160	1.080–1.246	<0.0001*

^*^ Means p<0.05.

### 3.2 Impact of surgical approaches on CL

Subgroup analysis was further performed to compare the effect of surgical approaches on the management of CL, including minimally invasive surgery group (laparoscopic surgery and robot‐assisted laparoscopic surgery) and open surgery group. The results of the subgroup analysis are shown in Table 3. Compared with patients with CL after open surgery, patients after laparoscopic surgery had shorter onset time (2.4 ± 1.3 vs. 3.4 ± 1.4, *p* = 0.015), less maximum drainage volume (411.5 ± 272.9 vs. 831.3 ± 812.6, *p* = 0.026) and shorter time to oral intake (0.88 ± 1.6 vs. 3.4 ± 3.9, *p* = 0.011).

**TABLE 3:** Comparison between patients with chyle leak in the minimally invasive surgery and open surgery groups.

Factors	Total (*n* = 44)	Minimally invasive surgery (*n* = 18)	Open surgery (*n* = 26)	*p* value
Time to onset (days)	3.0 ± 1.5 (1–6)	2.4 ± 1.3 (1–5)	3.4 ± 1.4 (1–6)	0.015*
Time to oral intake (days)	2.4 ± 3.4 (0–14)	0.88 ± 1.6 (0–6)	3.4 ± 3.9 (0–14)	0.011*
Time to resolution (days)	5.9 ± 7.7 (1–49)	4.0 ± 2.1 (1–8)	7.1 ± 9.6 (1–49)	0.24
Retention time of drainage tube (days)	11.7 ± 10.5 (3–59)	8.6 ± 4.3 (3–14)	14.2 ± 12.8 (4–59)	0.070
Drainage output on the first day after surgery (mL)	340.7 ± 466.8 (20–2670)	260.5 ± 239.3 (10–910)	396.2 ± 572.8 (20–2670)	0.58
Maximum drainage output during the treatment (mL)	677.6 ± 674.0 (46–3612)	411.5 ± 272.9 (46–1256)	831.3 ± 812.6 (105–3612)	0.026*
Postoperative hospitalization (days)	13.9 ± 12.6 (4–70)	9.7 ± 3.7 (4–15)	16.5 ± 15.5 (6–70)	0.071

^*^ Means p<0.05.

### 3.3 Impact of CL on postoperative hospitalization and oncological outcome

**FIGURE 4:**
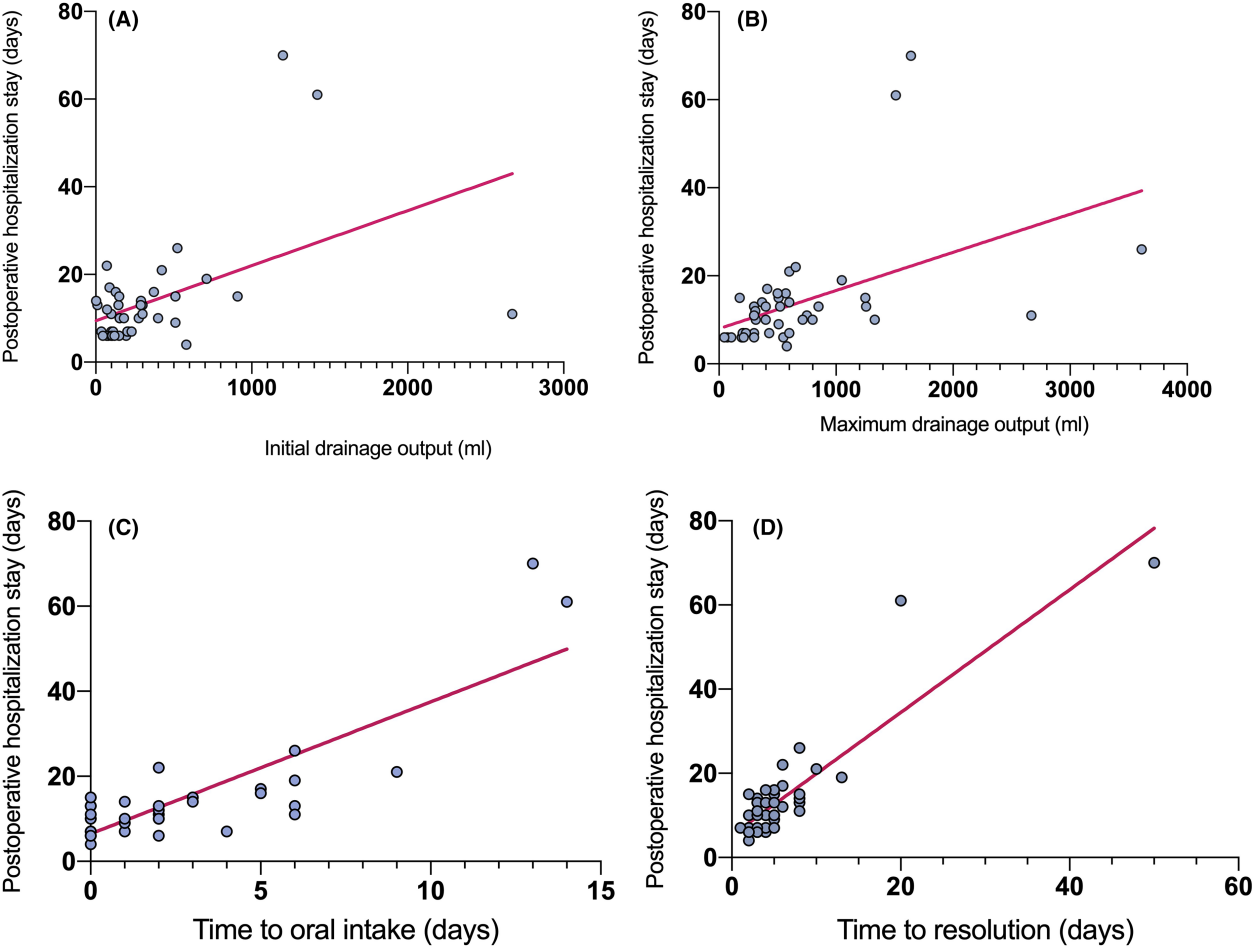
Correlations of postoperative hospitalization stay with (A). Initial drainage output, (B) Maximum drainage output during the treatment, (C) Time to oral intake, (D) Time to resolution. (A) Postoperative hospitalization stay (days) = 0.01256* Initial drainage output +9.449 (*n* = 44, *r*_s_ = 0.362, *p* = 0.0157); (B) Postoperative hospitalization stay (days) = 0.008666 * Maximum drainage output +7.995 (*n* = 44, *r*_s_ = 0.578, *p* < 0.0001); (C) Postoperative hospitalization stay (days) = 3.103 * Time to oral intake +6.475 (*n* = 44, *r*_s_ = 0.650, *p* < 0.0001); (D) Postoperative hospitalization stay (days) = 1.458 * Time to resolution +5.294 (*n* = 44, *r*_s_ = 0.693, *p* < 0.0001).

The occurrence of postoperative CL significantly prolongs the postoperative hospitalization of patients (13.7 ± 12.5 vs. 8.7 ± 5.6, *p* < 0.0001). We further analyzed the impact of clinical factors related to CL on length of postoperative hospitalization (Table 4). Patients with Grade B/C, more drainage output, and longer fasting time have longer postoperative hospitalization. To further illustrate the relationship between CL and postoperative hospitalization, a correlation analysis was conducted on the drainage output on the first day after surgery (Figure 4A), the maximum drainage output (Figure 4B), the time to oral intake (Figure 4C), and the time to resolution (Figure 4D) with postoperative hospitalization days, which shows significant correlation between them.

**TABLE 4:** Impact of chyle leak on postoperative hospitalization.

Factors	n	Postoperative hospitalization (days)	*p* value
Chyle leak			<0.0001*
Yes	44	13.7 ± 12.5 (4–70)	
No	401	8.7 ± 5.6 (1–55)	
Grade			0.0001*
A	35	10.2 ± 4.2 (4–22)	
B/C	9	27.8 ± 22.0 (11–70)	
Drainage output on the first day after surgery			0.030*
≥500 mL	9	25.6 ± 23.6 (4–70)	
No	35	10.7 ± 4.4 (6–22)	
Maximum drainage output during the treatment			0.0034*
≥1000 mL	8	28.1 ± 23.7 (10–70)	
No	36	10.5 ± 4.5 (4–22)	
Time to onset (days)			0.038*
≤3 days	25	12.1 ± 12.6 (4–70)	
No	19	15.8 ± 12.3 (6–61)	
Time to oral intake			0.0002*
≤2 days	30	9.7 ± 3.9 (4–22)	
No	14	22.3 ± 19.1 (6–70)	

^*^ Means p<0.05.

**FIGURE 5:**
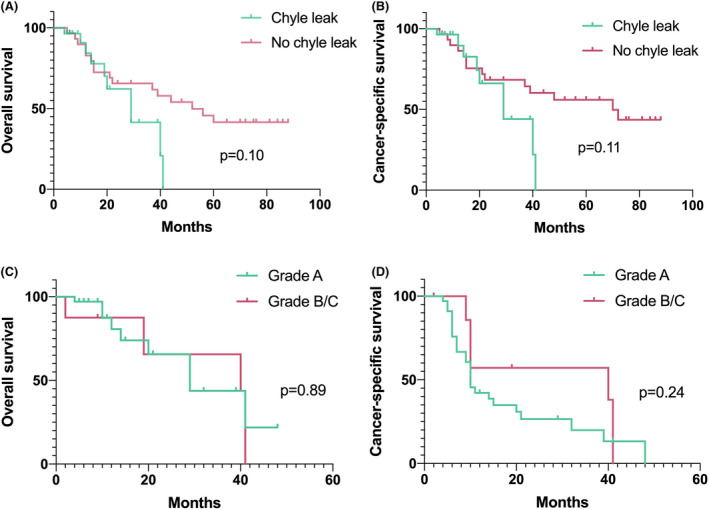
Kaplan Meier survival curves in overall survival or cancer‐specific survival of CL patients after RNAT (A) OS between CL group and non‐CL group; (B) CSS between CL group and non‐CL group; (C) OS between Grade A and Grade B/C in CL group: (D) CSS between Grade A and Grade B/C in CL group.

After PSM, the baseline data between the CL group and the non‐CL group did not show statistical differences (Supplementary Materials 1). Between the CL group and the non‐CL group, neither OS nor CSS showed statistical differences (Figure 5A,B). The patients who had CL were divided into two groups of Grade A and Grade B/C, and OS and CSS did not show statistical differences (Figure 5C,D).

## 4 DISCUSSION

Postoperative CL is a rare complication that occurs after urological surgeries, mainly in lymph node dissection for testicular cancer or renal cell carcinoma.^3^, ^6^ The incidence of CL in urological surgeries ranges from 0.77% to 15% depending on the type of surgery and the diagnostic criteria for CL.^3^, ^8^ RNAT is one of the most challenging urological surgeries,^15^ as it involves extensive resection of the renal vein or IVC, increasing a higher risk of disrupting the cisterna chyli. Persistent CL can result in hypoalbuminemia, lymphocytopenia, and electrolyte imbalance.^8^ However, there are currently no reports of CL after RNAT and its incidence, prevention, and clinical implications. Therefore, we analyzed patients with CL after RNAT in a large medical center to define the incidence and risk factors of CL after RNAT and determine its clinical implications.

The pathogenesis of postoperative CL involves the disruption of the cisterna chyli or the main lymphatic vessels,^3^, ^6^, ^16^ leading to the leakage of chyle fluid into the abdominal cavity. In addition, spontaneous small leakage of lymphatic vessels also contributes. When the drainage tube was removed, the patient developed CL, usually manifested by progressive abdominal distension and dyspnea, which was due to the accumulation of chyle in the abdominal cavity and compression of the diaphragm.

In kidney‐related surgery, CL is more prevalent in patients with left kidney involvement due to the location of the cisterna chyli and the main lymphatic vessels.^3^ The lumbar lymphatic vessels drain most of the lymph in the abdominal cavity and lower limbs, and ascend along the aorta and IVC into the peritoneum and then into the cisterna chyli.^9^ When extensive retroperitoneal resection or lymph node dissection is performed during left radical nephrectomy, the likelihood of its disruption is greatly increased. When RCC invades IVC, it necessitates surgical management of IVC. Due to the extension of the lumbar trunk along the abdominal aorta and IVC, the lymphatic vessels may be disrupted during the surgical management of IVC TT, which is one of the reasons that the incidence of CL in RNAT is higher than that in radical nephrectomy. We categorize TT into low Mayo grade and high Mayo grade, which represent different effects on IVC during surgical management. From the results of multivariate regression analysis, we indicate that patients with high Mayo grade TT are more prone to develop CL, as high Mayo grade TT means a larger surgical area and more surgical management of the IVC.

Although obtaining more lymph nodes can help confirm the presence of tumor invasion, it also means an increased risk of postoperative CL. This may be due to the disconnection or injury of lymphatic vessels during lymph node dissection, making it difficult to completely close them through electrocoagulation or ligation.^17^ In addition, due to the proximity of the RNAL surgical area to the cisterna chyli, although some patients did not receive lymph node dissection, CL also occurred. Adopting the following methods can help reduce postoperative CL. Firstly, for patients with tumors located on the left side, grade 3 or 4 TT, more caution should be exercised in handling the tissue around the cisterna chyli and lymphatic vessels, as these patients have a higher risk of developing CL. Careful ligation of non‐vascular strip‐like tissues reduces the risk of chyle leakage from 24% to 5.3% in patients with completely clamped lymphatic vessels during surgery.^18^ When ligating non vascular ducts, bundle by bundle ligation could be used. When the surgical scope reaches the high position of the main chyle cistern, titanium clips or Hem‐o‐lok are used to close the main and branches. Secondly, due to the difficulty of fully closing lymphatic vessels through electrocoagulation due to disconnection or injury, it is usually caused by increased lymphatic reflux after a decrease in pneumoperitoneum pressure. Therefore, it is more reliable to use ultrasonic knife, titanium clip, Hem‐o‐lok, or 4‐0 Prolene to close lymphatic vessels. Thirdly, in laparoscopic or robotic surgery, increasing pneumoperitoneum pressure during lymph node dissection helps to close lymphatic vessels and reduce leakage. Finally, due to the decrease in pneumoperitoneum pressure leading to an increase in lymphatic reflux, it is recommended to observe the presence of white fluid leakage in the surgical field after lowering the pneumoperitoneum pressure at the end of the surgery. It is also beneficial to use bioprotein glue to seal small lymphatic vessels after lymph node dissection. Within 1 week after surgery, for patients with risk factors for CL, it is recommended to extend fasting time and preventive dietary control after surgery to reduce the occurrence of CL.

The short‐term impact of CL on patients is reflected in the prolongation of postoperative hospitalization time.^4^ Although in this article, minimally invasive surgery has a higher postoperative drainage volume, shorter resolution time, and shorter fasting time, there is insufficient evidence to suggest that minimally invasive surgery has an advantage in‐hospital stay. We consider this because most patients receive short‐term relief through conservative treatment, even though CL seems more severe after open surgery.^5^ Interestingly, we found that the postoperative hospitalization days and maximum drainage volume seem to be positively correlated. This suggests that patients should be reminded to stay longer in the hospital after a significant increase in drainage is detected. In addition, through analysis of patients' OS and CSS, we did not find a significant effect of CL on patient outcomes, even at higher grades. Previous studies have revealed the same result, namely that CL is a complication that resolves with conservative treatment in most patients without compromising prognosis.^16^, ^19^, ^20^

There are still some limitations in this study. First, this is a single‐center retrospective study, and the research results may be influenced by data bias. Therefore, further data is needed to further validate the conclusions of this study in the future. Second, due to the small sample size and considering selection bias, we analyzed the risk factors for patients who experienced postoperative CL hospitalization for more than 14 days.

## 5 CONCLUSION

In retrospective analysis of patients with CL after RNAT surgery, we found that the risk factors were Mayo grade, side, operation approach, operation time and number of lymph nodes harvested, and the occurrence of CL significantly prolonged hospital stay, but had no effect on long‐term oncological outcomes.

## AUTHOR CONTRIBUTIONS

**Kewei Chen:** Conceptualization (equal); data curation (equal); formal analysis (equal); investigation (equal); methodology (equal); software (equal); supervision (equal); validation (equal); visualization (equal); writing – original draft (equal); writing – review and editing (equal). **Zhuo Liu:** Conceptualization (equal); formal analysis (equal); methodology (equal); project administration (equal); resources (equal); software (equal); supervision (equal); writing – original draft (equal); writing – review and editing (equal). **Yuxuan Li:** Formal analysis (equal); methodology (equal). **Xun Zhao:** Conceptualization (equal); data curation (equal); resources (equal); validation (equal). **Guoliang Wang:** Project administration (equal); resources (equal). **Xiaojun Tian:** Project administration (equal); resources (equal). **Hongxian Zhang:** Conceptualization (equal); formal analysis (equal). **Lulin Ma:** Conceptualization (equal); data curation (equal); funding acquisition (equal); methodology (equal); project administration (equal); resources (equal); validation (equal); visualization (equal). **Shudong Zhang:** Conceptualization (equal); formal analysis (equal); funding acquisition (equal); investigation (equal); project administration (equal); resources (equal).

## FUNDING INFORMATION

This research received the National Natural Science Foundation of China (No. 81972381 and No. 82173385).

## CONFLICT OF INTEREST STATEMENT

None.

## ETHICS STATEMENT

All patients have been given written informed consent and the study protocol was approved by the institute's committee on human research. This study protocol was reviewed and approved by the Peking University Third Hospital Medical Science Research Ethics Committee (M2021484).

## CONSENT FOR PUBLICATION

The use of clinical data in this study was authorized by each patient, and the patients agreed for the data to be published publicly.

## Supporting information


Data S1.

## Data Availability

The data used or analyzed in the current study are available from the corresponding author on reasonable request.
