# Supplementary material for: Prevention, incidence, and risk factors of chyle leak after radical nephrectomy and thrombectomy
Source: Cancer Med. 2023 Dec 20;13(1):e6858. doi: 10.1002/cam4.6858 (PMC10807595; doi:10.1002/cam4.6858)
Supplement: Supplementary file 1 — Data S1. [file CAM4-13-e6858-s001.docx]

**Supplementary Material**

**Supplementary Material 1** Baseline characteristics of patients undergoing RNAT before and after propensity matching

|  | Before propensity matching | | p value | After propensity matching | | p value |
| --- | --- | --- | --- | --- | --- | --- |
|  | Chyle leak group (n=44) | Non-chyle leak group (n=401) |  | Chyle leak group (n=31) | Non-chyle leak group (n=31) |  |
| Gender |  |  | 0.85 |  |  | $>$0.99 |
| Male | 31 (70.5%) | 277 (69.1%) |  | 21 (67.7%) | 21 (67.7%) |  |
| Female | 13 (29.5%) | 124 (30.9%) |  | 10 (32.3%) | 10 (32.3%) |  |
| Age (Years) | 56.8$\pm$12.7  (23-78) | 58.5$\pm$11.7  (20-83) | 0.40 | 57.0$\pm$11.7  (30-77) | 57.9$\pm$9.6  (37-74) | 0.73 |
| Weight (Kg) | 65.3$\pm$12.9  (43.5-100.0) | 67.8$\pm$11.9  (37.0-115.0) | 0.21 | 65.8$\pm$12.5  (47.0-100.0) | 68.7$\pm$12.2  (53.0-112.0) | 0.41 |
| Height (cm) | 166.1$\pm$7.5  (150.0-180.0) | 167.0$\pm$7.60  (143.0-188.0) | 0.48 | 166.6$\pm$7.9  (150.0-180.0) | 166.5$\pm$7.1  (150.0-179.0) | 0.98 |
| Body Mass Index (Kg  /m^2^) | 23.5$\pm$3.76  (16.7-33.0) | 24.2$\pm$3.57  (15.2-39.0) | 0.27 | 23.6$\pm$3.23  (18.8-33.0) | 24.7$\pm$3.30  (19.3-35.0) | 0.22 |
| Operation approach |  |  | 0.006* |  |  | $>$0.99 |
| Open | 26 (59.1%) | 173 (43.1%) |  | 18 (58.1%) | 18 (58.1%) |  |
| Laparoscopy | 7 (15.9%) | 162 (40.4%) |  | 6 (19.3%) | 6 (19.3%) |  |
| Robot-assisted laparoscopy | 11 (25.0%) | 66 (16.5%) |  | 7 (22.6%) | 7 (22.6%) |  |
| Laparoscopy to open |  |  | 0.22 |  |  | 0.51 |
| Yes | 7 (15.9%) | 40 (10.0%) |  | 4 (12.9%) | 7 (22.6%) |  |
| No | 37 (84.1%) | 361 (90.0%) |  | 27 (87.1%) | 24 (77.4%) |  |
| Mayo grade |  |  | 0.006* |  |  | $>$0.99 |
| 0 or 1 | 11 (25.0%) | 186 (46.4%) |  | 9 (29.0%) | 9 (29.0%) |  |
| 2, 3 or 4 | 33 (75.0%) | 215 (53.6%) |  | 22 (71.0%) | 22 (71.0%) |  |
| Ipsilateral adrenalectomy |  |  | 0.35 |  |  | 0.80 |
| Yes | 25 (56.8%) | 198 (49.4%) |  | 19 (61.3%) | 18 (58.1%) |  |
| No | 19 (43.2%) | 203 (50.6%) |  | 12 (38.7%) | 13 (41.9%) |  |
| Partial resection of IVC |  |  | 0.73 |  |  | 0.59 |
| Yes | 12 (27.3%) | 100 (24.9%) |  | 11 (35.5%) | 9 (29.0) |  |
| No | 32 (72.7%) | 301 (75.1%) |  | 20 (64.5%) | 22 (71.0%) |  |
| Lymph node dissection |  |  | 0.0006* |  |  | 0.12 |
| Yes | 30 (68.2%) | 165 (41.2%) |  | 21 (67.7%) | 15 (48.4%) |  |
| No | 14 (31.8%) | 236 (58.8%) |  | 10 (32.3%) | 16 (51.6%) |  |
| Lymph node harvest | 6.3$\pm$6.0  (n=30, range, 1-31) | 4.5$\pm$3.6  (n=165, range, 1-20) | 0.040* | 5.9$\pm$6.3  (n=21, range, 1-31) | 6.3$\pm$5.2  (n=165, range, 1-15) | 0.99 |
| Hemoglobin (g/L) | 115.9$\pm$18.7  (84-149) | 124.3$\pm$23.9  (40-232) | 0.011* | 113.9$\pm$17.5  (84-148) | 110.4$\pm$23.2  (72-150) | 0.51 |
| Albumin (g/L) | 38.1$\pm$6.2  (21-50) | 40.0$\pm$5.3  (19-51) | 0.17 | 38.6$\pm$5.2  (27-50) | 37.8$\pm$6.3  (24-49) | 0.56 |
| preoperative Scr ($\mu$mol/L) | 99.4$\pm$43.6  (45-304) | 102.8$\pm$82.1  (32-958) | 0.86 | 104.4$\pm$48.3  (52-304) | 97.9$\pm$37.6  (53-219) | 0.74 |
| postoperative Scr ($\mu$mol/L) | 120.5$\pm$75.8  (51-445) | 113.0$\pm$81.6  (34-875) | 0.55 | 123.3$\pm$116.0  (65-445) | 119.5$\pm$94.2  (34-519) | 0.84 |
| Side |  |  | 0.0001* |  |  | 0.79 |
| Left | 29 (65.9%) | 146 (36.4%) |  | 19 (61.3%) | 20 (64.5%) |  |
| Right | 15 (34.1%) | 255 (63.6%) |  | 12 (38.7%) | 11 (35.5%) |  |
| Tumor diameter (cm) | 9.3$\pm$3.8  (4.0-21.1) | 8.7$\pm$3.3  (1.5-20.0) | 0.40 | 9.6$\pm$4.0  (4.0-21.1) | 9.0$\pm$2.6  (4.5-14.7) | 0.47 |
| Hepatic vein invasion |  |  | 0.69 |  |  | $>$0.99 |
| Yes | 1 (2.3%) | 6 (1.5%) |  | 1 (3.3%) | 1 (3.3%) |  |
| No | 43 (97.7%) | 395 (98.5%) |  | 30 (96.7%) | 30 (96.7%) |  |
| Perinephric tissues invasion |  |  | 0.057 |  |  | 0.80 |
| Yes | 19 (43.2%) | 117 (29.2%) |  | 13 (41.9%) | 12 (38.7%) |  |
| No | 25 (56.8%) | 284 (70.8%) |  | 18 (58.1%) | 19 (61.3%) |  |
| Sarcomatoid differentiation |  |  | 0.23 |  |  | $>$0.99 |
| Yes | 4 (9.1%) | 64 (16.0%) |  | 4 (12.9%) | 3 (9.7%) |  |
| No | 40 (90.9%) | 337 (84.0%) |  | 27 (87.1%) | 28 (90.3%) |  |
| Pathological type |  |  | 0.20 |  |  | 0.93 |
| Clear cell carcinoma | 28 (63.6%) | 272 (67.8%) |  | 20 (64.5%) | 21 (67.7%) |  |
| Papillary cell carcinoma | 7 (15.9%) | 32 (8.0%) |  | 6 (19.4%) | 6 (19.4%) |  |
| Other | 9 (20.5%) | 97 (24.2%) |  | 5 (16.1%) | 4 (12.9%) |  |
| ASA classification |  |  | 0.70 |  |  | 0.36 |
| 1 | 2 (4.5%) | 22 (5.5%) |  | 1 (3.2%) | 3 (9.7%) |  |
| 2 | 34 (77.3%) | 320 (79.8%) |  | 24 (77.4%) | 25(80.6%) |  |
| 3 | 8 (18.2%) | 53 (13.2%) |  | 6 (19.4%) | 3 (9.7%) |  |
| 4 | 0 (0%) | 6 (1.5%) |  | 0 (0%) | 0 (0%) |  |
| Operation time (min) | 373.5$\pm$185.5  (91-1114) | 298.2$\pm$126.1  (60-995) | 0.0036* | 354.5$\pm$159.8  (91-873) | 377.6$\pm$132.3  (77-635) | 0.54 |
| Intraoperative hemorrhage (ml) | 1694$\pm$1730  (10-7000) | 985.8$\pm$1312  (5-10000) | 0.0008* | 1566$\pm$1684  (10-7000) | 1787$\pm$1913  (10-8800) | 0.54 |
